# Diagnostic Evaluation of Liver Fibrosis using B1-Corrected T1 Mapping and DWI-Based Virtual Elastography

**DOI:** 10.2174/0115734056401119250908130930

**Published:** 2025-09-23

**Authors:** Yuanqiang Zou, Jiaqi Chen, Jinyuan Liao

**Affiliations:** 1 Department of Radiology, The First Affiliated Hospital of GuangXi Medical University, Nanning, China

**Keywords:** Liver fibrosis, Magnetic resonance elastography, cirrhosis, tissue stiffness, T1 mapping, Liver biopsy, Fibrosis imaging

## Abstract

**Introduction:**

Liver fibrosis is a key pathological process that can progress to cirrhosis and liver failure. Although magnetic resonance elastography (MRE) is an established noninvasive method for fibrosis staging, its clinical application is limited by hardware dependence. The diagnostic utility of diffusion-weighted imaging-based virtual MRE (vMRE) and B1-corrected T1 mapping in liver fibrosis assessment remains to be further investigated.

**Methods:**

Forty rabbits were included in the final analysis: CCl_4_-induced fibrosis (n=33) and control (n=7). Following Gd-EOB-DTPA administration, DWI and T1 mapping sequences were executed at 5 and 10 minutes. Diagnostic efficacy and correlations of vMRE and T1 mapping in a rabbit liver fibrosis model were evaluated.

**Results:**

Rabbits were classified into three groups: Control (n=7), Nonadvanced fibrosis (F1-F2, n=20), and Advanced fibrosis (F3-F4, n=13). The AUC values for T1post_5min, T1post_10min, rΔT1_10min, and μdiff in distinguishing controls from nonadvanced and advanced fibrosis groups were (0.78, 0.82, 0.71), (0.82, 0.85, 0.77), and (0.62, 0.69, 0.74), respectively, with μdiff showing (0.90, 0.93, 0.66). A significant positive correlation existed between μdiff and liver fibrosis grade (r=0.534, p<0.001).

**Discussion:**

μdiff correlated well with fibrosis severity and effectively identified fibrotic livers, but showed limited ability to distinguish fibrosis stages, likely due to overlapping tissue stiffness. B1-corrected T1 mapping offered complementary functional information, with the 10-minute post-contrast time point providing the best staging performance, thereby enhancing the overall diagnostic value.

**Conclusion:**

Gd-EOB-DTPA-enhanced T1 mapping and DWI-based vMRE provide substantial noninvasive assessment of liver fibrosis.

## 1 INTRODUCTION

Liver fibrosis is a response to chronic liver damage caused by various factors, including persistent hepatitis, excessive alcohol use, cholestasis, and metabolic abnormalities [1-3]. It progresses to cirrhosis and can lead to liver failure [4]. While cirrhosis is often considered irreversible, studies indicate that advanced fibrosis can be reversible [5].

Thus, accurate evaluation of liver fibrosis is critical in clinical practice. Although liver biopsy is the gold standard for diagnosing liver fibrosis, it has certain disadvantages. These include sampling errors, observer interpretation variability, low tolerance for repeated procedures, and bleeding risks [6-9]. Consequently, liver biopsy is unsuitable for regular screening in individuals suspected of having liver fibrosis. Developing non-invasive indicators for detecting liver fibrosis is crucial.

Magnetic Resonance Elastography (MRE) is widely recognized as the most accurate noninvasive modality for fibrosis staging, with clinical studies reporting area under the curve (AUC) values as high as 0.95 for detecting advanced fibrosis [10-12]. It offers whole-liver coverage, low technical failure rate, and excellent reproducibility [13, 14]. However, MRE requires additional hardware and complex post-processing, which hinders its widespread clinical implementation [15, 16].

To overcome these limitations, Le Bihan *et al*. proposed a novel concept in 2017 virtual MRE (vMRE) based on intravoxel incoherent motion (IVIM)-derived DWI metrics [17]. By converting the apparent diffusion coefficient (ADC) into a shear modulus through calibration, vMRE enables estimation of liver stiffness without external vibration, offering high sensitivity to fibrosis-related microstructural changes [18, 19]. DWI acquired at high b-values is particularly sensitive to non-Gaussian diffusion behavior, thereby capturing microstructural complexity that underlies tissue fibrosis.

In contrast to vMRE, T1 mapping reflects the intrinsic longitudinal relaxation time of tissue, which depends on its physical, chemical, and biological properties. It can be performed with standard MRI equipment, requires no contrast agent or additional hardware, and is easy to standardize and quantify. Several studies have shown that T1 mapping correlates with liver fibrosis severity [20-22], yet results have been inconsistent, possibly due to physiological confounders such as liver perfusion, fat content, and B1 field inhomogeneity.

Given their complementary advantages T1 mapping’s sensitivity to tissue composition and vMRE’s capacity to quantify mechanical stiffness we hypothesize that evaluating both techniques in the same experimental cohort may clarify their respective diagnostic performance for liver fibrosis.

Therefore, the aim of this study was to evaluate and compare the diagnostic performance of T1 mapping and vMRE each applied independently in the non-invasive assessment of liver fibrosis. We employed a well-established carbon tetrachloride (CCl_4_)-induced rabbit model and used hepatocyte-specific contrast-enhanced MRI with Gd-EOB-DTPA to assess liver function and structure. The pharmacokinetics of Gd-EOB-DTPA in animal models has been extensively validated in prior studies [23, 24], supporting its application in preclinical fibrosis imaging.

## 2 MATERIALS AND METHODS

### 2.1 Animals and Model Construction

Animal experiments were approved by the Institutional Animal Care and Use Committee of Guangxi Medical University. This study adheres to internationally accepted standards for animal research, following the 3Rs principle. The ARRIVE guidelines were employed for reporting experiments involving live animals, promoting ethical research practices. Healthy New Zealand White rabbits (aged 6 months, weighing 2.5-3.0 kg, both genders) were obtained from the Experimental Animal Center of Guangxi Medical University. Animals were randomly divided into two groups: the liver fibrosis group (F1-F4) (n=33) and the normal control group (n=7). In the liver fibrosis group, a 50% CCl_4_ oil solution (1:1 mixture of 100% CCl_4_ and olive oil) was injected subcutaneously at the back of the neck, once or twice a week. Doses were 0.1 mL/kg for the first 3 weeks, 0.2 mL/kg from the fourth to sixth weeks, and 0.3 mL/kg for the seventh to tenth weeks [25]. Rabbits in the control group received the same dose and frequency of saline injections.

### 2.2 MRI Protocol

Before imaging, rabbits underwent a 12-h fasting period and were anesthetized using a small animal anesthesia machine. An abdominal belt was applied to stabilize the abdomen, and rabbits were positioned supine to reduce respiratory motion. T1 mapping was performed at 5, 6, 7, and 10 weeks post-injection using a 3T MRI system (MAGNETOM Prisma, Siemens Healthineers, Germany) equipped with an 8-channel rabbit-specific coil (Jiangsu Zhongzhi Medical Technology Co., Ltd., China).

Liver T1 mapping was conducted before and at 5, 10, and 15 minutes after Gd-EOB-DTPA (Primovist; Bayer Healthcare, Germany) administration using a 3D spoiled gradient echo sequence. To improve T1 quantification accuracy, a B1 map covering the entire liver was acquired prior to T1 mapping (acquisition time: 10 s) and applied to correct flip angle deviations. This approach follows the concept of B1 inhomogeneity correction as described by Yoon *et al*., which improves the accuracy of volumetric liver T1 mapping following hepatocyte-specific contrast enhancement [26].

Imaging parameters included: TR = 5.79 ms, TE = 2.46 ms, voxel size = 3.6 × 2.5 × 4.7 mm^3^. B1-corrected T1 maps were generated at each post-contrast time point. Diffusion-weighted imaging (DWI) was performed using a free-breathing single-shot echo-planar imaging (ss-EPI) sequence with eleven b-values (0–2000 s/mm^2^) in three orthogonal directions. Additional parameters were: TR/TE = 5000/49 ms, FOV = 223 × 150 mm^2^, matrix = 74 × 50, slice thickness = 3 mm, gap = 0.3 mm, acceleration factor = 2, and scan time = 7 min 56 s.

### 2.3 Imaging Analysis

Images were analyzed in a double-blind mode independently by two physicians specializing in abdominal imaging. Any discrepancies were resolved through discussion.

### 2.4 Acquisition of T1 Mapping Quantitative Parameters

**Fig. (1):**
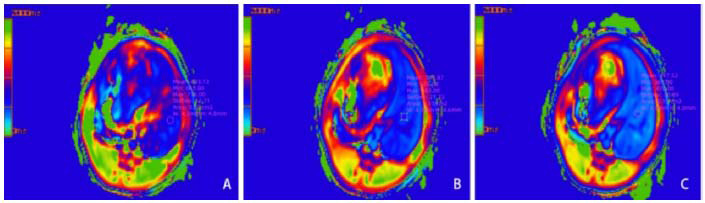
T1pre is depicted on the pre-contrast imaging **(A)**, while T1post_5min **(B)** and T1post_10min **(C)** images were acquired at 5- and 10-min post-contrast administration, respectively.

On anatomical MR images of the liver parenchyma, a region of interest (ROI) of 0.15 cm^2^ was outlined, avoiding vessels, artifacts, and liver boundaries. These ROIs were applied to the T1 map for precise T1 relaxation time measurements on a pixel-by-pixel basis. The average T1 values were analyzed. The reduction rate of T1 relaxation time, rΔ%, was calculated using the formula: rΔ% = (T1pre - T1post) / T1pre × 100%, where T1pre and T1post are the T1 times before and 5 and 10 min post-gadoxetic acid injection, respectively (Fig. **1**).

### 2.5 Post-processing of vMRE and Acquisition of Stiffness Values

**Fig. (2A-C):**
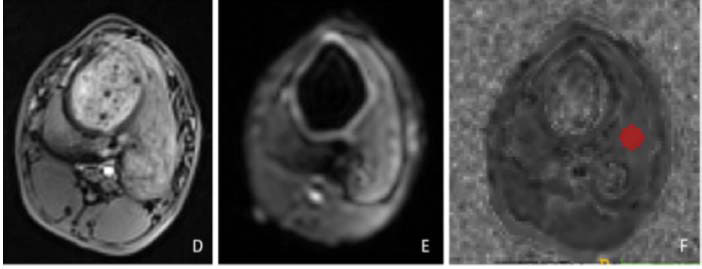
T1WI, DWI, and vMRE from left to right.

Quantification of vMRE stiffness was completed offline using MATLAB R2021a (USA). Previous studies [18, 27] indicated that only two B-value sequences are necessary for vMRE images. Thus, we selected shifted ADCs with B-values of 200 and 1000 s/mm^2^ (sADC). The formula sADC = ln (S200/S1000) / (200 - 1000) was used to calculate sADC in mm^2^/s, where S200 and S1000 represent the image signals at b-values of 200 s/mm^2^ and 1000 s/mm^2^, respectively. Stiffness values were determined using μdiff = α·sADC + β, where μdiff represents the diffusion-based shear modulus (in kPa), reflecting tissue stiffness, with the scaling factor α = −9.8 and shift factor β = 14.0, as previously determined in a calibration step by Le Bihan *et al*. [17, 27] Imaging results were reviewed by two physicians specializing in abdominal imaging through a double-blind method. In case of disagreement, they reviewed the images together to reach a consensus. A third senior radiologist with 20 years of experience was consulted for unresolved disagreements. The VOI was outlined in the left liver lobe using ITKsnap software (http://www.itksnap.org), focusing on liver parenchyma while avoiding hepatic vessels, bile ducts, and liver margins. The VOI volume was 0.33 cm^3^. The software extracted all voxels within the ROI to calculate the average μdiff (kPa) (Fig. **2**).

### 2.6 Pathological Examination

After MRI, rabbits were euthanized within 2 h. Liver tissues were fixed in formalin, embedded in paraffin, and sectioned at 6 micrometers for histological evaluation using Masson's trichrome stain. At least two sections per rabbit were examined by a certified pathologist with over 15 years of experience. Liver fibrosis was staged using the METAVIR system: F0 (no fibrosis), F1 (fibrous portal expansion), F2 (few bridges or septa), F3 (numerous bridges or septa), and F4 (cirrhosis) [28, 29].

### 2.7 Statistical Analysis

Data analysis was performed with GraphPad Prism version 9.5 and MedCalc version 22.0. The Shapiro-Wilk test was used to assess data distribution normality. For normally distributed data, means ± standard deviations are reported, and group differences were analyzed using the t-test. For non-normally distributed data, medians (interquartile range P25, P75) are presented, and the Mann-Whitney U test was used to compare group variances. Spearman's rank correlation was used to explore the relationship between μdiff and hepatic fibrosis severity. To identify significant differences in T1 mapping and μdiff values, the appropriate statistical test was applied. Receiver operating characteristic (ROC) curves were used to highlight significant group differences, computing the area under the curve (AUC). Optimal cutoffs were identified by the maximum Youden's index, along with sensitivity and specificity. Data with a P-value < 0.05 were deemed statistically significant.

## 3 RESULTS

### 3.1 Pathological Results

**Fig. (3):**
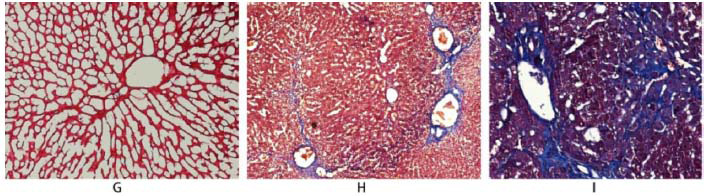
Masson’s trichrome staining of rabbit liver tissue under light microscopy (10× magnification). (**A**) Control group: normal hepatic architecture with no fibrosis. (**B**) Non-advanced fibrosis group: portal fibrosis with limited septa formation. (**C**) Advanced fibrosis group: extensive septa with architectural distortion, with or without nodular changes.

Our initial goal was to include at least 10 rabbits in each of the four stages of liver fibrosis. However, seven rabbits succumbed to CCl_4_ intolerance, two died during anesthesia, and one was excluded due to an unsuccessful contrast injection. In addition, the progression of fibrosis stages was not strictly time-dependent, and Stages 3 and 4 were less commonly observed. Furthermore, although we initially expected that rΔT1_5min, T1post_10min, rΔT1_10min, and μdiff would show significant differences across all fibrosis stages, no significant differences were observed between F1 and F2 or between F3 and F4 (data not shown). Therefore, based on both fibrosis severity and parameter distribution, we combined F1 and F2 into a Non-advanced fibrosis group, and F3 and F4 into an Advanced fibrosis group. Ultimately, the animals were categorized into three groups: the control group (n = 7), the Non-advanced fibrosis group (n = 20), and the Advanced fibrosis group (n = 13) (Fig. **3**).

### 3.2 Comparison of Quantitative Parameters of T1 Mapping in Different Liver Fibrosis Groups

As shown in Table **1**, there were significant differences in the three T1 mapping quantitative parameter values (T1post_5min, T1post_10min, rΔT1_10min) among the Control, Non-advanced fibrosis (F1-F2) and Advanced fibrosis (F3-F4) groups (*P*<0.05). However, no significant differences (P > 0.05) were found in T1 pre and rΔT1_5 min across the three groups. Following post hoc two-sample comparisons utilizing the Least Significant Difference (LSD) test, no statistically significant differences were found in T1pre measurements between the Control group and the Non-advanced fibrosis group, the Control group and the Advanced fibrosis group, or the Non-advanced fibrosis group and Advanced fibrosis group (P > 0.05). In contrast, all pairs except for the Control group versus the Nonadvanced fibrosis group demonstrated statistically significant differences in T1post_5min and T1post_10min values (*P* < 0.05). Notably, the relative change in T1 relaxation time at 5 min (rΔT1_5min) and 10 min (rΔT1_10min) showed marked disparities only between the Non-advanced fibrosis and Advanced fibrosis groups (*P*<0.05); these differences were not statistically significant across any other pair-wise comparisons (P > 0.05).

### 3.3 ROC Curve Analysis of Quantitative T1 Mapping

**Fig. (4):**
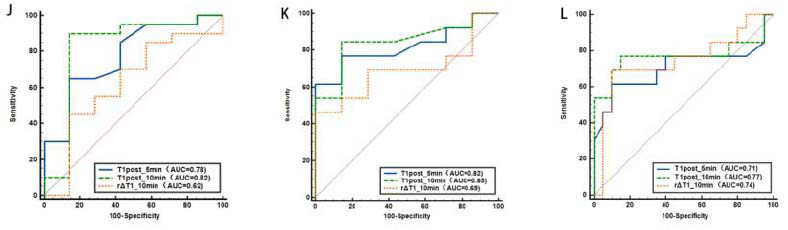
ROC curves of T1post_5min, T1post_10min, and rΔT1_10min for differentiating liver fibrosis stages. Panel (**A**) shows the ROC curves for Control *vs.* Nonadvanced fibrosis, panel (**B**) for Control *vs.* Advanced fibrosis, and panel (**C**) for Nonadvanced *vs.* Advanced fibrosis.

The AUCs for T1post_10min in differentiating the three groups were 0.82, 0.85, and 0.77, respectively, higher than those for T1post_5min (AUC=0.78, 0.82, and 0.71) overall, and rΔT1_10min (AUC=0.62, 0.69, and 0.74) had lower diagnostic value, as indicated in (Table **2** and Fig. **4**).

### 3.4 Group μdiff Value Measurements and Differences between Groups

**Fig. (5):**
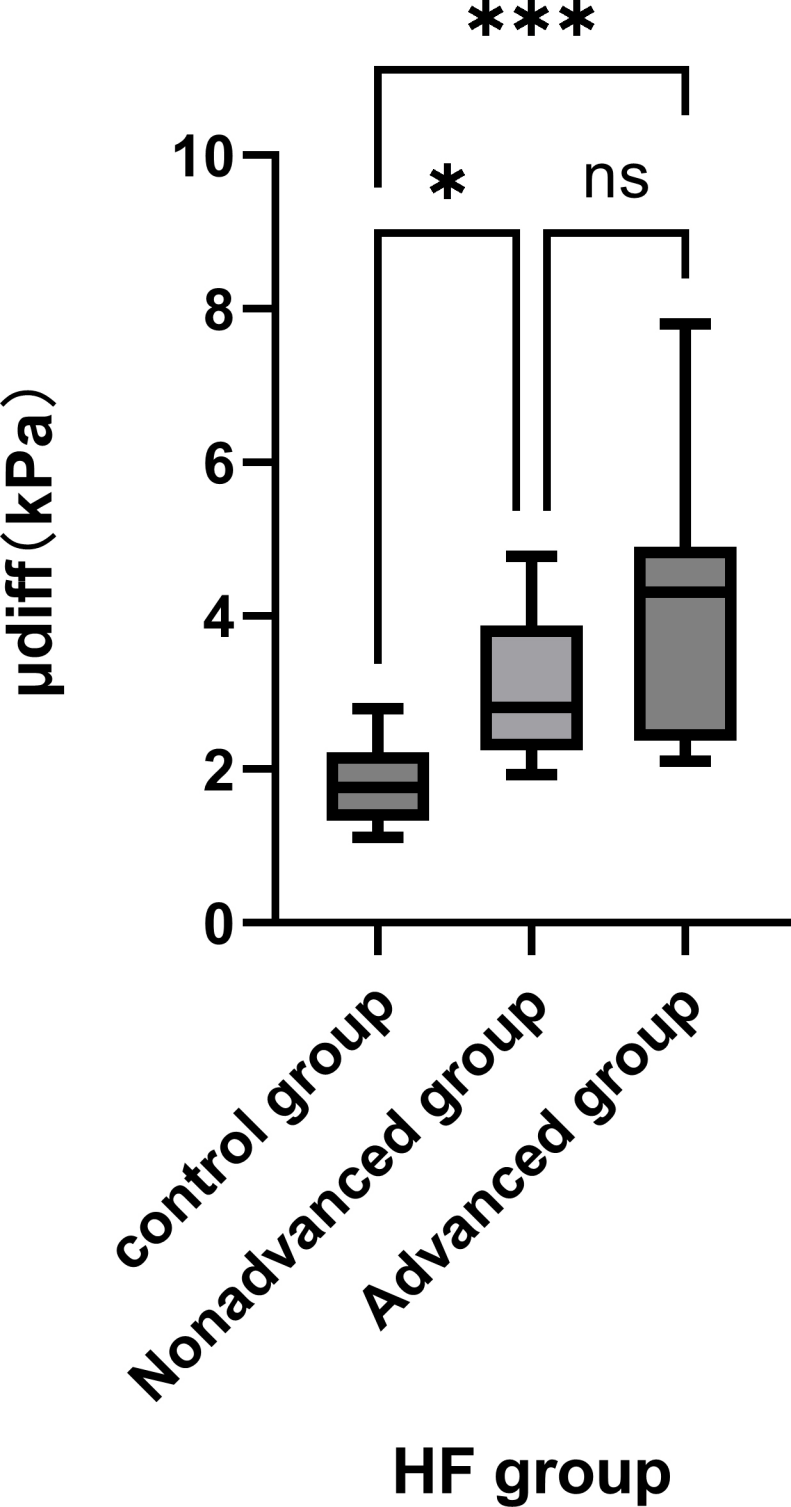
Measurement of μdiff values for respective rabbit groups.

µdiff measurements across the groups showed a non-normal distribution. The Kruskal-Wallis test revealed significant differences in μdiff values among the three groups (*P*<0.001). Further comparisons found the μdiff value in the Advanced fibrosis group was significantly higher than in the Control group (*P*<0.001). Similarly, the Non-advanced fibrosis group had a significantly higher μdiff value than the Control group (*P*<0.001). However, the difference between the Advanced and Non-advanced fibrosis groups was not significant (*P*>0.05) (Table **3** and Fig. **5**).

### 3.5 Correlation Analysis between µdiff Value and Liver Fibrosis Grouping

**Fig. (6):**
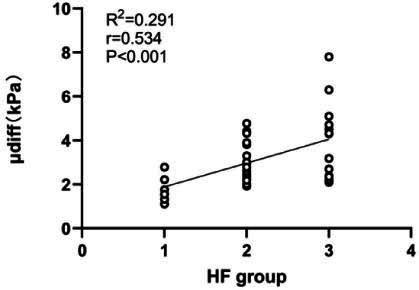
Spearman correlation analysis showed a moderate positive correlation between μdiff values and liver fibrosis grouping.

The µdiff measurements show a moderate positive correlation (r = 0.53, *P* < 0.001) with fibrosis groups (Fig. **6**).

### 3.6 ROC Curve Analysis of μdiff Measurements

**Fig. (7):**
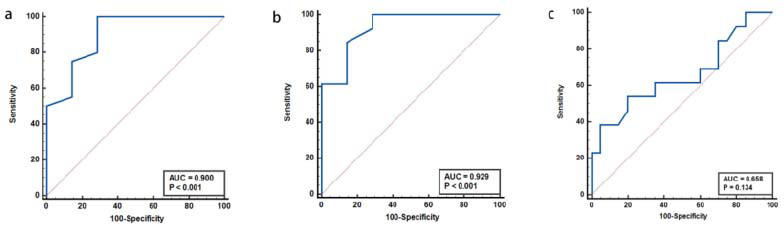
ROC curves of μdiff for differentiating liver fibrosis stages. ( **A** ) Control *vs.* Non-advanced fibrosis; ( **B** ) Control *vs.* Advanced fibrosis; ( **C** ) Advanced *vs.* Non-advanced fibrosis. The μdiff value exhibited superior diagnostic efficacy in distinguishing the Control group from both the Non-advanced and Advanced fibrosis groups, while showing reduced performance in differentiating between the Non-advanced and Advanced stages.

The AUC for μdiff in distinguishing between the Control group and Non-advanced fibrosis group was 0.90, and between the Control and Advanced fibrosis groups was 0.93. These values exceeded those of quantitative parameters T1post_10min (AUC= 0.82, 0.85) and T1post_5min (AUC= 0.78, 0.82) derived from enhanced MRI T1 mapping. However, μdiff showed limited effectiveness in differentiating between Non-advanced and Advanced fibrosis (AUC=0.66, *P*>0.05) (Table **4** and Fig. **7**).

## 4 DISCUSSION

Liver fibrosis is pathologically characterized by excessive extracellular matrix (ECM) accumulation, particularly collagen, which narrows the extracellular space and increases tissue complexity [3]. As cirrhosis substantially raises the risk of hepatocellular carcinoma (HCC) and liver-related mortality [30], timely diagnosis and intervention before progression to cirrhosis are critical.

In this study, we investigated the feasibility of using B1-corrected EOB-DTPA-enhanced T1 mapping parameters and μdiff values derived from diffusion-weighted virtual MR elastography (vMRE) to noninvasively assess liver fibrosis in a CCl_4_-induced rabbit model. Among the quantitative T1 mapping parameters, T1post_5min, T1post_10min, and rΔT1_10min significantly differed across control, non-advanced (F1–F2), and advanced (F3–F4) fibrosis groups (*P* < 0.05). In contrast, T1pre and rΔT1_5min did not show statistically significant group differences (P > 0.05). The μdiff values also varied significantly across the three groups (*P* < 0.05), with a moderate positive correlation with histological fibrosis severity (r = 0.534, *P* < 0.001).

Notably, while the diagnostic performance of μdiff was high in distinguishing fibrotic from non-fibrotic livers (AUC = 0.90 for non-advanced fibrosis and 0.93 for advanced fibrosis), its ability to discriminate between non-advanced and advanced fibrosis stages was limited (AUC = 0.66). We consider that this limitation may be due to the fact that fibrosis progression at the microstructural level does not correspond linearly to histological staging, resulting in overlapping tissue stiffness between F2 and F3–F4 stages. Since vMRE estimates stiffness based on diffusion characteristics rather than direct histopathological architecture, it may not fully capture subtle differences in extracellular matrix remodeling. This underperformance suggests that although vMRE is sensitive for detecting the presence of fibrosis, it may lack sufficient granularity for accurate staging an important consideration for clinical translation. These limitations highlight the need for cautious interpretation of μdiff-based results and suggest that further refinement of the parameter or its application strategy may be necessary.

Previous studies have demonstrated the potential of T1 mapping in human liver fibrosis assessment [31, 32]. Our results are consistent with Zhou * et al*. [ 23], who found no significant group difference in T1pre values with Gd-EOB-DTPA. However, contrast-enhanced T1 mapping at delayed phases particularly at 10 minutes showed better differentiation of fibrosis stages, in line with the findings of Haimerl *et al.* [33]. The decline in T1 values after contrast administration reflects impaired hepatocellular function and altered contrast uptake, reinforcing its potential as a noninvasive surrogate for fibrosis grading.

Jung *et al*. [34] found high consistency in liver fibrosis stage evaluation between MRE and vMRE through retrospective imaging data analysis. This indicates the stability and effectiveness of vMRE, with the potential to replace MRE. In our rabbit model, vMRE-generated μdiff values accurately diagnosed early and late liver fibrosis (AUC=0.90 and AUC=0.93, respectively) but were less effective in differen-tiating between these stages (AUC=0.66). We also noted a moderate positive correlation between μdiff values and liver fibrosis grade, aligning with findings from Kromrey *et al*. [35].

## 5 LIMITATIONS

This study has several important limitations. First, only a CCl_4_-induced fibrosis model was used, which may not fully represent other etiologies such as nonalcoholic steatohepatitis (NASH), viral hepatitis, or alcoholic liver disease. Second, due to animal mortality and uneven fibrosis progression, the sample size across fibrosis stages was imbalanced, potentially affecting statistical power and the generalizability of our results. Third, no external validation or comparison with human liver data was performed, limiting clinical extrapolation [18]. Finally, confounding histopathological changes including inflammation, steatosis, and iron deposition may influence T1 mapping and μdiff values, complicating direct interpretation.

## CONCLUSION

In conclusion, quantitative parameters from enhanced T1 mapping with Gd-EOB-DTPA and μdiff values from vMRE enhance diagnostic and staging capabilities for liver fibrosis in CCl_4_-induced models. Notably, vMRE was found more effective in assessing liver fibrosis than quantitative parameters from T1 mapping. Future research should address the technical limitations of vMRE, explore fibrosis of various etiologies, validate findings in human cohorts, and consider combining multiple quantitative MRI biomarkers to improve diagnostic accuracy and staging resolution.

## Figures and Tables

**Table 1:** To compare the mean values of T1Pre, T1post_5min, r∆T1_5min, T1post_10min, and r∆T1_10min across the three groups, a one-way Analysis of Variance (ANOVA) followed by the Least Significant Difference (LSD) post-hoc test was employed, with a significance level of 0.05.

Groups (Mean ± SD)	T1Pre	T1post_5min	rΔT1_5min	T1post_10min	rΔT1_10min
Control group (*n*=7)	718.43±54.93a	338.71±26.25b	0.56±0.08ab	252.29±41.58b	0.68±0.06ab
Non-advanced fibrosis group (*n*=20)	721.95±68.16a	364.80±28.03b	0.58±0.06b	274.50±29.78b	0.69±0.05a
Advanced fibrosis group (*n*=13)	732.23±101.14a	398.08±51.01a	0.53±0.07a	339.31±64.94a	0.63±0.07b
*F*	0.094	6.473	2.382	10.993	4.585
*p*	0.911	0.004**	0.106	0.000**	0.017*

Note: * *p*<0.05, ** *p*<0.01;LSD: letter notation.

**Table 2:** Efficiency of T1 mapping parameters in differentiating groups.

**Parameters**	**Control *vs*. Nonadvanced fibrosis**	**Control *vs*. Advanced fibrosis**	**Advanced fibrosis *vs*. Nonadvanced fibrosis**
T1post_5min			
AUC (95% CI)	0.78(0.57 - 0.91)	0.82(0.59 - 0.95)	0.71(0.52 - 0.85)
Cutoff value	350	350	398
Sensitivity (%)	65	76.92	61.54
Specificity (%)	85.71	85.71	90
T1post_10min			
AUC (95% CI)	0.82(0.63 - 0.94)	0.85(0.62 - 0.97)	0.77(0.59 - 0.90)
Cutoff value	250	250	290
Sensitivity (%)	90.00	84.62	76.92
Specificity (%)	85.71	85.71	85
rΔT1_10min			
AUC (95% CI)	0.62(0.42 - 0.80)	0.69 (0.45 - 0.88)	0.74 (0.56 - 0.88)
Cutoff value	0.69	0.58	0.63
Sensitivity (%)	45	46.15	69.23
Specificity (%)	85.71	100	90

**Table 3:** Measurement of stiffness values (µdiff) for different rabbit groups.

**M (P25,P75)**	**μdiff**
Control group (*n*=7)	1.760(1.3,2.2)
Non-advanced fibrosis group (*n*=20)	2.800(2.2,3.9)
Advanced fibrosis group (*n*=13)	4.310(2.4,4.9)
Kruskal-Wallis H-test statistic (H)	13.127
*p*	0.001**

* *p*<0.05 ** *p*<0.01.

**Table 4:** Efficiency of µdiff values in different rabbit groups.

**Parameters**	**Control *vs.* Nonadvanced fibrosis**	**Control *vs.* Advanced fibrosis**	**Advanced fibrosis *vs.* Nonadvanced fibrosis**
μdiff			
AUC (95% CI)	0.90 (0.72 - 0.98)	0.93(0.64 - 0.98)	0.66(0.47 - 0.81)
Cutoff value	1.76	1.76	3.9
Sensitivity (%)	100	100	53.85
Specificity (%)	71.43	71.43	80

## Data Availability

The data and supportive information are available within the article.

## LIST OF ABBREVIATIONS

MRE: Magnetic Resonance Elastography
AUC: Area Under the Curve
ADC: Apparent Diffusion Coefficient
DWI: Diffusion-weighted imaging
ROC: Receiver operating characteristic
ECM: Excessive extracellular matrix
HCC: Hepatocellular carcinoma
